# Gradient Enhanced Strain Hardening and Tensile Deformability in a Gradient-Nanostructured Ni Alloy

**DOI:** 10.3390/nano11092437

**Published:** 2021-09-18

**Authors:** Xinlai An, Weikang Bao, Zuhe Zhang, Zhouwen Jiang, Shengyun Yuan, Zesheng You, Yong Zhang

**Affiliations:** Herbert Gleiter Institute of Nanoscience, School of Materials Science and Engineering, Nanjing University of Science and Technology, Nanjing 210094, China; xinlaian@njust.edu.cn (X.A.); baoo@njust.edu.cn (W.B.); zzh_njust@163.com (Z.Z.); 116116001370@njust.edu.cn (Z.J.); 912116150208@njust.edu.cn (S.Y.)

**Keywords:** gradient nanostructure, plasticity, strain hardening, microscale tension, mechanical properties

## Abstract

Gradient-nanostructured material is an emerging category of material with spatial gradients in microstructural features. The incompatibility between gradient nanostructures (GNS) in the surface layer and coarse-grained (CG) core and their roles in extra strengthening and strain hardening have been well elucidated. Nevertheless, whether similar mechanisms exist within the GNS is not clear yet. Here, interactions between nanostructured layers constituting the GNS in a Ni alloy processed by surface mechanical rolling treatment were investigated by performing unique microtension tests on the whole GNS and three subdivided nanostructured layers at specific depths, respectively. The isolated nanograined layer at the topmost surface shows the highest strength but a brittle nature. With increasing depths, isolated layers exhibit lower strength but enhanced tensile plasticity. The GNS sample’s behavior complied more with the soft isolated layer at the inner side of GNS. Furthermore, an extra strain hardening was found in the GNS sample, leading to a greater uniform elongation (>3%) as compared to all of three constituent nanostructured layers. This extra strain hardening could be ascribed to the effects of the strain gradients arising from the incompatibility associated with the depth-dependent mechanical performance of various nanostructured layers.

## 1. Introduction

Gradient materials are a new category of material in which certain intrinsic microstructural characteristics and/or properties, such as grain/precipitate size, chemical composition, magnetism, are distributed in gradient along the depth [1,2,3,4,5,6,7,8,9,10,11,12,13,14,15]. Gradient metals have exhibited great potentials for broad engineering applications due to enhanced properties of high strength, considerable ductility, and fatigue resistance, as compared to their homogeneous coarse-grained (CG) counterparts [5,6,7,16,17,18,19]. For metals, the microstructural gradients are readily achieved and tailored, and therefore have attracted broad attention over the past decade [5].

One of the critical advantages displayed by gradient metals is the unusual combinations of high strength and ductility [1,2,3,4,8,17,20,21,22]. For example, Fang et al. [2] engineered a gradient variation on the surface of CG Cu by surface mechanical grinding treatment (SMGT). The gradient-structured Cu offers a yield strength of 129 MPa, twice as high as CG Cu, while retaining the ductility [2]. Furthermore, Wu et al. [4] utilized surface mechanical attrition treatment (SMAT) to achieve a sandwich sheet structure in an interstitial-free (IF) steel characterized by gradient nanostructures (GNS) on two sides of a CG core. An extra strain hardening was found to lead to extraordinary ductility during tensile tests. The measurements for mobile dislocation density indicate the presence of strain gradient together with multiaxial stress states in the gradient structures, which promotes dislocation interactions and generation of geometrically necessary dislocations (GNDs) [4,23,24,25]. The present research focus has relied on the incompatibility between gradient-nanostructured layers and CG cores, and the resultant additional strengthening and strain hardening from the gradient structures [4,10,16,22,26,27,28,29], nonetheless, whether similar phenomena exist between neighboring nanostructured layers with distinct microstructural features remains to be addressed.

Essentially, the gradient-nanostructured layers on the surface of the gradient samples could also be considered as a composite consisting of several nanostructured layers with distinct microstructural characteristics and mechanical performance. Very recently, Cheng et al. [3] fabricated gradient-nanotwinned Cu plates with various structural units through electrodeposition. A series of tensile tests present that the gradient-nanotwinned Cu sample exhibit superior combinations of strength and ductility as compared to any of the nanotwinned units. Further microstructure observations and large-scale atomistic simulations revealed that the unprecedented mechanical properties originate from the so-called bundles of concentrated dislocations (BCDs), which are formed as a result of the strain gradient induced by the gradient variations in twin size in the nanoscale [3].

Therefore, it is imperative to understand the incompatibility between neighboring nanostructured layers in the gradient-structured metals, and the role of the gradients within the surface gradient-nanostructured layers playing in promoting the strength and strain hardening during tensile loading. In the present study, we tend to address this issue on a GNS of a Ni alloy processed by surface mechanical rolling treatment (SMRT) [19,30]. Compared with previous mechanical surface treatment methods, such as SMAT or SMGT, SMRT can generate a more homogeneous structure-refined layer at the length of rod with a smoother surface (Ra < 0.20 μm) because it can stably control the rolling reduction of each pass. Meanwhile, the lubricating oil not only takes away the processing heat to avoid the thermal recovery in refined grains, but also forms an oil film between the ball and the rod to avoid introducing pollution. Typically, the GNS and microstructure-refined layers produced by SMRT are much thicker due to much higher accumulated strains [19]. Considering the depth span of each nanostructured layer with representative microstructural features are typically on the order of ~50–100 μm [6,19,30,31], microtension specimens were laser-machined at three specific depth spans characterized by various microstructural features and mechanical response. Furthermore, microtension specimens with gauge width covering 0–300 μm were also laser-machined to determine the overall mechanical properties of the whole gradient-nanostructured layer. A direct comparison on the mechanical response was used to reveal the extra strain hardening arising from the gradient microstructures.

## 2. Experimental Methods

**Figure 1:**
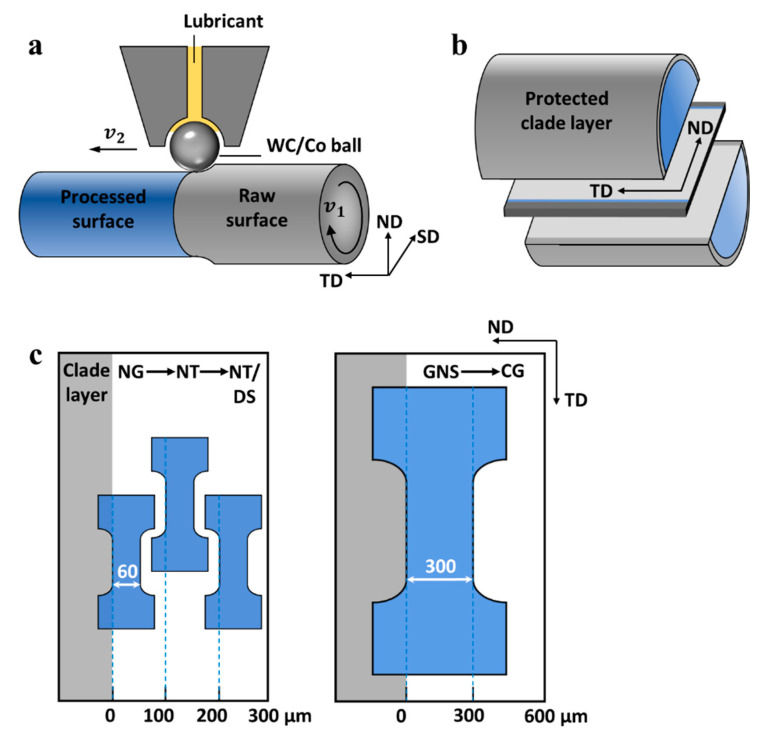
Schematic illustrations of (**a**) the SMRT set-up and the defined specimen coordinate system (shear direction (SD), transversal direction (TD), normal direction (ND)), and (**b**,**c**) sampling positions for the NG, NT, NT/DS, and GNS specimens.

The material utilized for SMRT processing is a polycrystalline Ni alloy with a nominal composition of 22.7 Cr, 13.8 W, 1.05 Mo, 1.01 Fe, 0.94 Co, 0.44 Al, 0.42 Mn, 0.38 Si, 0.08 C, and Ni in bal. (in wt.%). The alloy sample was cut into cylinders with a diameter of 10 mm and a length of 160 mm, and then homogenized at 1222 °C for 1 h followed by water-quenching. A homogeneous CG microstructure with an average grain size of ~110 μm was achieved. In the SMRT processing (Figure 1a), the as-received Ni alloy rod rotated at a velocity of *v*_1_ with respect to a WC/Co alloy ball, which was enforced into the rod surface with a nominal penetration depth of 20 μm. Meanwhile, the WC/Co alloy ball displaced along the transversal direction (TD) (i.e., longitudinal axis of rod) from one end to the other with a velocity of *v*_2_. Liquid lubricating oil was fed between the WC/Co ball and rod as a coolant to reduce the transient temperature rise associated with the localized plastic deformation. In order to facilitate the micro tensile tests at various depths, the SMRT process was repeated eight times with the same procedure to maximize the thickness of the gradient-nanostructured layer.

The microstructure characterization of the SMRT samples was performed on the shear direction (SD)-normal direction (ND) plane (Figure 1a), i.e., cross-sectional plane, by means of scanning electron microscope (SEM) and transmission electron microscope (TEM). The cross-sectional sample for SEM observations was mechanically polished and then electro-polished with an electrolyte consisting of 10% perchloric acid and 90% ethanol at −25 °C. Zeiss Auriga SEM was utilized to obtain the overall morphology of GNS via electron channeling contrast (ECC) imaging at 20 kV. The FEI Tecnai 20 was utilized for detailed characterizations and statistical measurements on the sizes of the GNS. Sample slices for TEM characterizations were taken parallel to the cross-sectional plane of the SMRT sample, and then mechanically polished down to ~50 μm thick. The transparent area in the TEM sample was obtained by electropolishing in a Struers Tenupol-5 twin-jet polishing unit, using an electrolyte containing 15% perchloric acid and 85% ethanol, at a temperature of −20 °C and a voltage of 20 V.

The microhardness tests were performed on the same plane (SD-ND plane) as that for microstructural characterizations. A series of indentations were conducted at various depths of the SMRT sample on a Qness Q10 A+ microhardness tester with a load of 50 g and a loading period of 10 s. The microhardness at each depth was averaged from at least three indentation tests. The uniaxial tensile properties of nanostructured layers at various depths were determined by means of a micro-tensile tester at a strain rate of 3 × 10^−4^ s^−1^. Figure 1b,c demonstrates the procedure for fabricating the micro tensile specimens. First, a protective layer of Ni was electrodeposited on the surface of the SMRT sample, and a slice of 500 μm in thickness was machined parallel to the TD-ND plane (Figure 1b). The slice was further mechanically ground to ~60 μm with SiC papers, and finished with a mirror-like surface by the same electropolishing process for SEM observations. Lastly, dog-bone-shaped micro tensile specimens with a gauge section of 250 μm in length, 60 μm in width, and 60 μm in thickness were machined on the longitudinal section with the loading direction parallel to the TD direction by means of a commercial femtosecond laser micromachining system (FemtoLAB, Workshop of Photonics), as shown in Figure 1c. The ultrashort pulse duration (~280 fs) and high pulse energy fluence (4.8 J/cm^−2^) of the laser made the material ionized before the heat energy dissipated into the surrounding material, thereby avoiding the thermal effect on microtensile specimens [32]. To ensure measurements of mechanical properties on representative microstructures at various depths, the micro tensile specimen was cut at three specific depths with gauge width covering regions of 0–60 μm, 100–160 μm, and 200–260 μm, respectively. Furthermore, microtensile specimens with gauge width covering 0–300 μm were also laser-machined.

## 3. Results and Discussion

**Figure 2:**
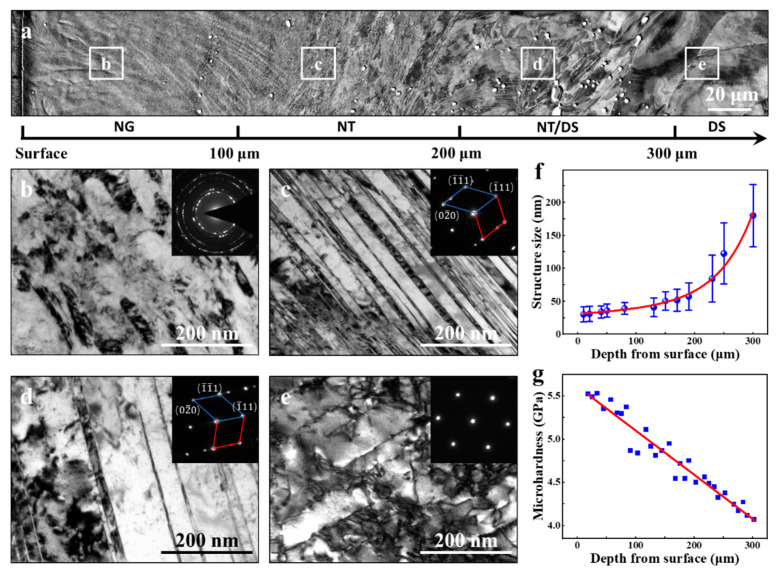
(**a**) A typical cross-sectional SEM image of the SMRT Ni alloy sample. (**b**–**e**) Typical bright-field cross-sectional (SD-ND) TEM images of NG, NT, NT/DS, and DS structures indicated in (**a**), respectively. Different regions are identically marked in (**a**). The insets are the corresponding SAED patterns obtained by using an aperture with a diameter of 200 nm. (**f**,**g**) Variations of average structure size and microhardness along with the depth from the surface.

Figure 2a shows a representative cross-sectional SEM image of the SMRT sample, showing varied microstructural features with increasing the distance to the surface. In the region from the surface to ~100 μm in depth, the deformation layer exhibits a uniform contrast, and the original coarse-grained grain boundaries (GBs) are not distinguishable, indicating a significant grain refinement due to the large strain and strain rate exerted on the surface [6,33,34,35]. In the depth region of 100–300 μm, the microstructure is characterized by a high density of planar defects, which tend to comply with the shear direction. The density of the planar defects becomes lower with increasing depth. In the region with a distance of >300 μm from the surface, although certain amounts of planar defects within grain interiors remain visible, the original GBs appear to be easily identified. The distinct microstructural features observed at various depths of the SMRT sample indicate a formation of GNS in the depth span of 0–300 μm. The present observations are consistent with previous results obtained in other materials, such as Ni [6,36,37], Cu [2,38], Al alloy [21,39], and Ni alloy [12,40,41], subjected to surface mechanical processing.

Figure 2b–e displays the bright-field TEM micrographs of typical microstructures at the aforementioned various regions, respectively. In the topmost surface layer (0–100 μm), the microstructure is characterized by slightly elongated nanograins (NG) with long axis inclined with the SD. Figure 2b shows an example of the bright-field TEM image of elongated NG taken at region b in Figure 2a. Statistical measurements show that the average transverse axis size at the depth of 20 μm is ~30 nm. This size is comparable to those obtained in the surface layers of Ni [6,37], Ni-Cr-Fe alloy [12,40] processed by various surface processing techniques. The selected area electron diffraction (SAED) pattern displays a ringlike feature, suggesting that the NG may have random crystallographic orientations. In the depth of 100–300 μm, TEM observations indicate that the planar defects revealed by SEM-ECC imaging are mainly composed of nanotwins (NT), as evidenced by the inset SAED patterns of Figure 2c,d corresponding to the microstructures at regions c and d in Figure 2a. The average twin/matrix (T/M) lamellar thickness increases from 40 nm at region c to 85 nm at region d, indicating an increasing trend of T/M lamellar thickness with increasing depths. With increasing depths, the density of NTs is decreased as the strains and strain rates are lowered in the depth span of 200–300 μm. Dislocation structures (DS) become prominent in this region, leading to a mixed microstructure of NT and DS (NT/DS). In the region with a depth of >300 μm, such as region e in Figure 2a, considerable amounts of DS can be detected in grain interiors, as shown in the TEM micrograph taken aligned with the [101] zone axis (Figure 2e). The predominant DS in the depth span of >300 μm of the present Ni alloy with low SFE suggests that the gradient strain rates and strains diminish to be small values at such distances to the processed surface. The rapid vanish of strain rates and strains with increasing depth agrees well with other observations of the low shear strains and strain rates at the depth >300 μm in SMGT-processed Ni [36]. The occurrence of deformation twinning as the dominant deformation mechanism at the depth of 100–300 μm can be ascribed to two factors. First, the low SFE of the present Ni-Cr-W alloy [42,43] facilitates deformation twinning as a major deformation mode. Furthermore, the high strain rate and strain exerted on the surface of the materials accompanied with the SMRT processing [19,30] also promote the tendency of the deformation twinning to accommodate the plastic deformation, leading to higher density of NT and/or smaller T/M lamellar thickness [34,35]. Another feature of the GNS is that there exists a conspicuous transition of the microstructure from NT to NG when approaching the surface of the SMRT sample. This indicates that deformation twinning plays a critical role in refining grains into the nanoscale in the present Ni-Cr-W alloy with low SFE via high strain-rate deformation [35]. Essentially, the refined NG could be envisaged as a result of the interactions between dislocations and twin boundaries at the nanoscale [34,35]. Similar fragmentation processes of NT in materials with various SFEs subjected to plastic deformation at high strain rates and cryogenic temperatures have been frequently observed in SMAT/SMGT Cu [38,44], DPD Cu [45,46], and Cu-Al alloys [47].

Sizes of microstructural features located between 0–300 μm depth are obtained by statistical measurements on a series of TEM photographs taken at various depths. The gradient variations of the short axis sizes of characteristic structures are plotted in Figure 2f. Overall, the characteristics sizes of the nanostructures increase with increasing the distance to the topmost surface, i.e., a gradient microstructure in terms of the sizes is generated accompanied with gradient distributions of strain rates and strains exerted on the surface by means of SMRT processing [19,30]. The average size of NG closest to the surface is as small as ~25 nm, and when approaching the original CG region, the mean T/M lamellar thickness at the depth of 300 μm increases up to ~180 nm. Furthermore, it is seen that within the top 100 μm-thick layer, the NG sizes are well below 50 nm, indicating a thicker nanograined layer as compared to SMGT processed Cu (~50 μm) [38] and Ni (~80 μm) [6]. The depth region of 100–300 μm can be divided into two distinct regions in terms of the T/M lamellar thickness. In the depth of 100–200 μm, the mean T/M lamellar thickness increases slightly with the average T/M lamellar thickness remained to be around 50 nm. In the depth of 200–300 μm, a steep increase of T/M lamellar thickness is exhibited with the average size increases from ~65 nm at 200 μm depth to ~180 nm at 300 μm depth. The preliminary hardness profile obtained below the processed surface (Figure 2g) illustrates a decreased microhardness from ~5.5 GPa at the topmost surface layer to ~4.1 GPa at the depth of 300 μm. The decreased microhardness is consistent with the increase on the sizes of the microstructural characteristics. It is evident that a depth-dependent gradient microstructure consisting of three distinct regions with various microstructural characteristics is produced on the processed surface, which enables the machining of micro tensile specimens at three specific depths for assessing the mechanical response of each representative microstructure.

**Figure 3:**
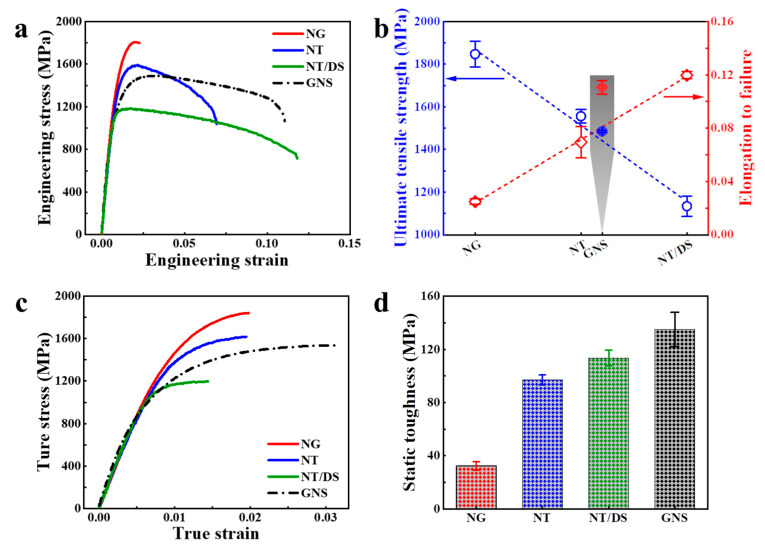
Comparisons between the GNS specimen and the isolated NG, NT, and NT/DS specimens: (**a**) engineering tensile stress-strain curves; (**b**) variation of ultimate tensile strength and elongation to failure; (**c**) true tensile stress-strain curves; (**d**) static toughness.

Figure 3a exhibits the engineering stress-strain curves obtained by micro tensile tests for the GNS and the three isolated structural layers (NG, NT, NT/DS). The curves of the isolated layers indicate that the tensile stresses and the tensile elongations vary substantially with microstructural changes. The strength of the NG layer is as high as 1848 ± 60 MPa because of the most severe grain refinement at the surface. The strengths of the sub-surface NT and the internal NT/DS layers decrease to 1556 ± 32 MPa and 1134 ± 48 MPa, respectively, due to the increased microstructural size. Meanwhile, after severe surface deformation, the grain refinement and the dislocation density in all the three microstructural layers are close to saturation, limiting further intragrain dislocation storage. As a consequence, the isolated layers by themselves exhibit negligible strain hardening and limited uniform elongations (approximately 1.44–1.97%) during the tensile tests. After the initiation of localized necking, the specimens with isolated microstructural layers behave rather differently. The NG specimen catastrophically fractures at a minor elongation of only 2.5%. By contrast, the NT and NT/DS specimens break at much greater elongations of 6.9% and 12%, respectively. Figure 3b shows that the elongation to failure increases and the ultimate tensile strength decreases both in a linear manner with increasing depth from surface (from NG to NT/DS). These strength-uniform elongation values are in good agreement with those specimens with comparable characteristic sizes in the literatures [48,49,50,51,52,53], indicating the microtensile tests can display the intrinsic properties of the specimens. For instance, Fan et al. obtained a high strength of 1990 MPa on bulk nanocrystalline Ni–Fe alloy (average grain size d = 23 nm) [52], which was similar to the grain size and strength of the NG specimen in the present work. 

When the three microstructural constituents are combined together, the GNS specimen exhibits an ultimate tensile strength of approximately 1485 MPa. Figure 3b shows the tensile strength is in agreement with the value estimated by the rule of mixture, and close to that of the intermediate NT layer. However, the elongation to failure of the GNS specimen (approximately 11%) is much greater than that of the NT layer, and only slightly lower than that of the isolated NT/DS layer (Figure 3b). This indicates that the tensile stabilities of both the NG and NT layers are substantially enhanced to the same level of the NT/DS layer when they constitute a gradient structure. According to the report of Zhao et al. [54] on relationships between specimen dimensions and mechanical properties, the specimens are designed with proportional gauge dimensions to overcome the issue for sample size effect. Therefore, the enhancement in tensile stabilities is credible and is not artificially caused by the larger specimen dimensions.

To demonstrate the extra strain hardening arising from the gradient structure, Figure 2c displays the true stress-strain curves prior to necking initiation for the GNS sample and the isolated microstructure layers. It is evident that the strain hardening of the NG, NT, and NT/DS samples decrease to a negligible level soon after yielding. However, the GNS specimen exhibits a continuous increment in the flow strength to a plastic strain over 3%, which is obviously greater than all of its microstructural components. Such a high strain hardening is not relevant to the elastic-plastic transition, because it is maintained to strains at which the strain hardening has completely diminished in the isolated NG layer. Therefore, it is believed that the extra strain hardening is an inherent feature of the gradient structure, which will be discussed below.

Figure 3d compares the static toughness represented by the integral area below the entire tensile curve for each specimen. The static toughness increases from 32 MPa for the isolated NG specimen, to 113 MPa for the isolated NT/DS specimen. It is noted in Figure 3d that the static toughness of the GNS specimen (135 MPa) is even greater than that of all the isolated specimens.

**Figure 4:**
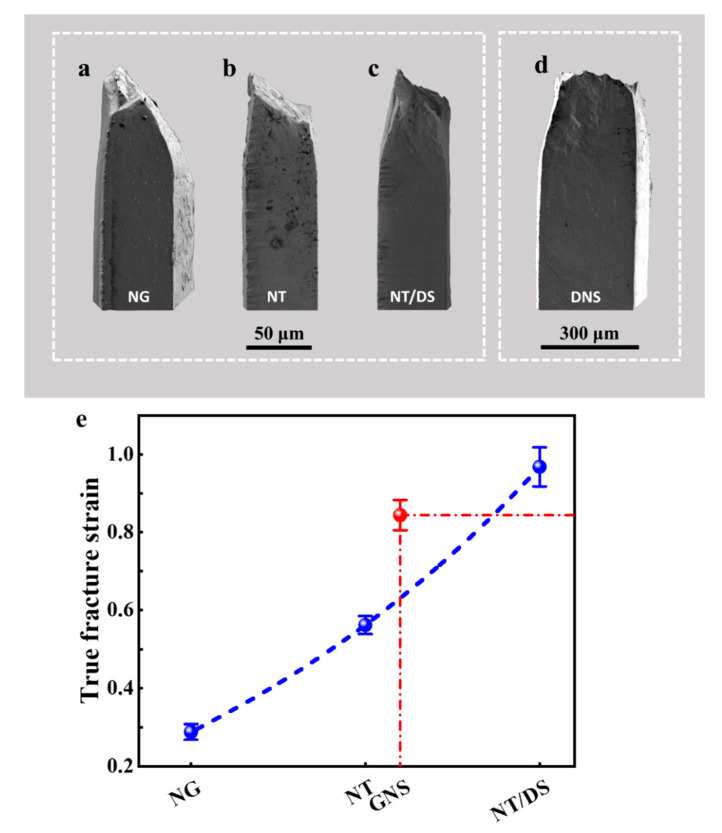
(**a**–**d**) Lateral morphology of the fractured NG, NT, NT/DS, and GNS specimens, respectively. (**e**) Variation of the true fracture strain of aforementioned specimens.

**Figure 5:**
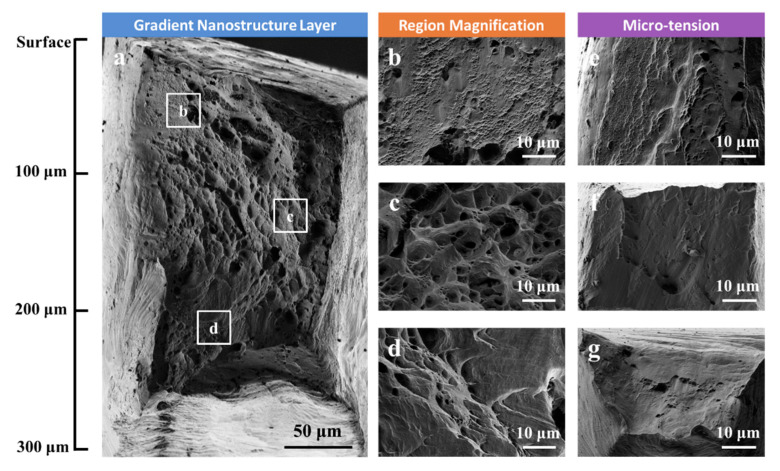
(**a**) Fracture surface of the GNS specimen. (**b**–**d**) Magnified images of the marked regions in (**a**), indicating NG, NT, and NT/DS structures, respectively. (**e**–**g**) Fracture surfaces of the isolated NG, NT, and NT/DS specimens after microtension.

Figure 4a–d displays side view observations on the fractured tensile specimens, which shows more intuitively the degree of tensile plasticity and localized necking. The isolated NG specimen (Figure 4a) fractured catastrophically in a shear fracture manner along an inclined plane after limited necking. In combination with the negligible post-necking elongation during the tensile test (Figure 3), this indicates that a slanted localized shear band probably nucleated shortly after the plastic yielding and eventually developed to a main crack. Similar to the NG specimen, the isolated NT specimen (Figure 4b) and NT/DS specimen (Figure 4c) are also characterized by inclined fracture planes. However, the magnitudes of localized necking prior to fracture of the NT and NT/DS specimens are much more remarkable than that of the NG specimen, a sign of increasing postnecking tensile plasticity from NG to NT/DS. When the three layers are combined together, the GNS specimen appears to fracture in a normal manner with the fracture surface almost vertical to the tensile axis, as shown in Figure 4d. Substantial necking also develops before fracture. It is worth noting that the NT/DS side exhibits much more evident necking than the NG side. To quantify the difference in tensile deformability of the isolated samples and the GNS sample, the true fracture strain, defined as ln(*A*_0_/*A*) where *A*_0_ and *A* are original and minimum cross-sectional areas after fracture, respectively, was determined for each specimen. Figure 4e shows that the true fracture strain increases from 28.8% for the isolated NG sample to 56.2% for the isolated NT sample and 96.8% for the isolated NT/DS sample. The GNS sample combining the three layers exhibits a true fracture strain of 84.4%, much higher than the isolated NG and NT samples, and only slightly lower than the isolated NT/DS sample. Careful SEM examinations on the deformed surface of the GNS sample did not reveal any cracks or voids on the surface NG layer. This suggests that the NG layer, although being rather brittle under separate tension (Figure 3), plastically deforms in concert with the other two layers in the GNS sample. Figure 5a exhibits an SEM image of the fracture surface of the GNS specimen. The typical fracture appearances of the NG, NT, and NT/DS regions in the GNS specimen are magnified and shown in Figure 5b–d, respectively. For comparison, the fracture surfaces of the isolated NG, NT, and NT/DS tensile specimens are also shown in Figure 5e–g, respectively. For the GNS sample, it is evident that the fracture morphology varies substantially in different microstructural regions. The outer NG and NT/DS layers are characterized by inclined shear fracture with arc-shaped dimples (Figure 5b,d), analogous to the isolated NG and NT/DS specimens (Figure 5e,g), whereas the intermediate NT layer fractures by normal fracture with almost equiaxial opening dimples with an average diameter of 5 μm (Figure 5c), which is rather different from the shear fracture without obvious dimples in the isolated NT specimen (Figure 5f). Based on these observations, it is expected that the failure of the GNS sample initiates by microvoid nucleation and coalescence in the intermediate NT layer while the surface NG and NT/DS layers remain intact. When the center crack extends to the surface, the NG and NT/DS layers fracture by shearing, like they are isolated layers. Therefore, the outer fracture appearances are similar to those of the isolated NG and NT/DS specimens. 

According to the results of the microtensile tests, the yield strength decreases gradually from NG, to NT and to NT/DS layer (Figure 3a,b). Therefore, in the GNS, when the soft NT/DS layer plastically yields, the hard NG layer is still in the elastic deformation stage. Such a layer-by-layer progressive yielding behavior plays an important role in the GNS in enhancing the strength-plasticity synergy. The GNS exhibits extra strain hardening and better uniform elongation compared to all of its constituent layers (Figure 3c). The underlying mechanisms can be discussed as follows. First, since the plastic deformation of the GNS sample occurs firstly in the soft NT/DS layer and propagates progressively to the hard NG layer, a plastic strain gradient is developed. To accommodate the plastic strain gradient, extra geometrically necessary dislocations (GNDs) should be incorporated correspondingly [55]. The generated GNDs interact and tangle with mobile dislocations to further raise dislocation storage capability [3,4,56]. Wu et al. proposed that these dislocation activities can effectively promote the dislocation accumulation near the elastic-plastic deformation boundaries, and induce nonlocal strengthening and significantly contribute to the observed extra hardening [4]. The extraordinary strain hardening is put forward to arise from the hetero-deformation-induced stress [57]. The pile-up of GNDs produces back stress in the soft layer and oppositely induces forward stress in the hard layer. The coupled back stress and forward stress set up long-range internal stresses in opposite directions, effectively contributing to the extra hardening in the GNS [22,58,59]. Another major factor for extraordinary strain hardening may be attributed to multiaxial stresses, which can explain the prolonged tensile elongation of the GNS sample [4,56]. The plastic strain gradient leads to progress on necking behavior from the NT/DS layer to the NG layer as well. The mutual constraint between the mechanically different microstructural layers leads to stress state changes. When the shrinkage of necking layers (surface NG and NT/DS) is constrained by the stable layer (internal NT), lateral stresses between the layers are formed. Therefore, the applied uniaxial tensile stress is converted to complex multiaxial stresses. With increasing strain, multiaxial stresses in the samples lead to the activation of additional slip systems and promote more dislocation activity, which consequently starts the extra strain hardening process in the late deformation stage [4,56].

## 4. Conclusions

In this work, a GNS was generated on the surface of a Ni alloy processed by SMRT, and microtensile tests were performed on the whole GNS layer and three subdivided layers, respectively. A direct comparison on the mechanical response of various samples was made to reveal the extra strain hardening induced by the effects of the strain gradients within the GNS. The main conclusions can be drawn as follows:

1. A gradient-nanostructured layer induced by the severe plastic deformation during SMRT was generated on the surface of a CG Ni alloy, with a thickness of 300 μm. The GNS is composed of three subdivided layers characterized by NG (0–100 μm), NT (100–200 μm), and a mixed structure of NT/DS (200–300 μm). The formation of GNS in a large depth span enables the micromachining of tensile specimens at various depths for assessing the mechanical response of each representative microstructure.
2. NG layer on the topmost surface (0–60 μm) of the GNS shows the highest strength but catastrophic fracture right after necking. By contrast, the subdivided nanostructured layers at larger depths exhibit lower strengths but enhanced tensile plasticities. The tensile curves of the GNS specimens show that when the isolated layers are combined together, the tensile ductility is comparable to that of the inner soft isolated layer characterized by NT/DS structures. This indicates that the tensile stabilities of both the NG and NT layers are substantially enhanced to the same level of the ductile NT/DS layer when they constitute a gradient structure.
3. A closer examination on the tensile true stress-strain curve of GNS specimens indicates that GNS induced an enhanced strain hardening, which leads to a much better uniform elongation as compared to all of three constituent layers. This extra strain hardening was ascribed to the effects of the strain gradients associated with the depth-dependent mechanical performance of various nanostructured layers.

## Data Availability

The data presented in this study are available on reasonable requests from the corresponding author.
